# An analysis of the strategic plan development processes of major public organisations funding health research in nine high-income countries worldwide

**DOI:** 10.1186/s12961-020-00620-x

**Published:** 2020-09-18

**Authors:** Cristina Morciano, Maria Cristina Errico, Carla Faralli, Luisa Minghetti

**Affiliations:** 1 Research Coordination and Support Service, Istituto Superiore di Sanità, Viale Regina Elena, 299, 00161 Rome, Italy; 2 National Centre for Disease Prevention and Health Promotion, Istituto Superiore di Sanità, Rome, Italy

**Keywords:** Health research, Research plan, Health research system, Research priority-setting

## Abstract

**Background:**

There have been claims that health research is not satisfactorily addressing healthcare challenges. A specific area of concern is the adequacy of the mechanisms used to plan investments in health research. However, the way organisations within countries devise research agendas has not been systematically reviewed. This study seeks to understand the legal basis, the actors and the processes involved in setting research agendas in major public health research funding organisations.

**Methods:**

We reviewed information relating to the formulation of strategic plans by 11 public funders in nine high-income countries worldwide. Information was collected from official websites and strategic plan documents in English, French, Italian and Spanish between January 2019 and December 2019, by means of a conceptual framework and information abstraction form.

**Results:**

We found that the formulation of a strategic plan is a common and well-established practice in shaping research agendas across international settings. Most of the organisations studied are legally required to present a multi-year strategic plan. In some cases, legal provisions may set rules for actors and processes and may establish areas of research and/or types of research to be funded. Commonly, the decision-making process involves both internal and external stakeholders, with the latter being generally government officials and experts, and few examples of the participation of civil society. The process also varies across organisations depending on whether there is a formal requirement to align to strategic priorities developed by an overarching entity at national level. We also found that, while actors and their interactions were traceable, information, sources of information, criteria and the mechanisms/tools used to shape decisions were made less explicit.

**Conclusions:**

A complex picture emerges in which multiple interactive entities appear to shape research plans. Given the complexity of the influences of different parties and factors, the governance of the health research sector would benefit from a traceable and standardised knowledge-based process of health research strategic planning. This would provide an opportunity to demonstrate responsible budget stewardship and, more importantly, to make efforts to remain responsive to healthcare challenges, research gaps and opportunities.

## Background

Advances in scientific knowledge have contributed greatly to improvements in healthcare, but there have been claims that health research is not adequately addressing healthcare challenges. These concerns are reflected in the increasing debate over the adequacy of the mechanisms used to plan investment in health research and ensure its optimal distribution [1–5].

Over recent decades, methods and tools have been produced in order to guide the process of setting the health research agenda and facilitate more explicit and transparent judgment regarding research priorities. There is no single method that is considered appropriate for all settings and purposes, yet it is recognised that their optimal application requires a knowledge of health needs, research gaps and the perspectives of key stakeholders [6–10].

A number of studies have described initiatives to set health research agendas. Several articles refer to experiences focusing on specific health conditions, for example, those undertaken under the framework of the James Lind Alliance [11]. There are also reviews of disparate examples of research agenda-setting in low- and middle-income countries [12, 13] as well as in high-income countries (HICs) [14]. These initiatives were highly heterogeneous with regard to their promotor (public organisations, academics, advocacy groups, etc.), the level of the research system (global, regional, national, sub-national, organisational or sub-organisational) and the scope of the prioritisation process (broad themes or specific research questions).

However, there are no studies that have specifically investigated the way large public organisations in HICs devise their research agendas and to what extent this is linked to regulations and organisational setup. In 2016, Moher et al. reported on how research funders had addressed recommendations to increase value and reduce waste in biomedical research [15]. Within this framework, they provided a general overview of setting the overall agenda in a convenient sample of six public funders of health research. They also affirmed the need for a “*periodic survey of information on research funders’ websites about their principle and methods used to decide what research to support*” [15]. At the same time, Viergever et al. identified the 10 largest funders of health research in the world and recommended further study of their priority-setting processes [16].

Given this context, we wished to provide an updated and thorough description of the way public funders of research in HICs devise their research agenda. We therefore analysed the regulatory framework for the actors and processes involved in developing the strategic plan in 11 major English and non-English speaking public research funders across 9 HICs worldwide.

## Methods

### Strategic planning

Our analysis focused on the development of the strategic plan, or strategic planning, at organisational level as a crucial step in the setting of the research agenda by the organisation. By the term ‘setting the research agenda’, we meant the whole-organisation research management planning cycle, which may encompass multiple decision-making level (organisational, sub-organisational, research programme level, etc.) actors and funding flows.

Strategic planning has been defined in social science as a “*deliberative, disciplined effort to produce fundamental decisions and actions that shape and guide what an organization (or other entity) is, what it does, and why*” [17].

The strategic plan is assumed to be the final outcome of the strategic planning process, in which priority-setting is the key milestone. It is therefore expected that the research priorities of the organisation will be included. Depending on mandate, priorities could be related to research topics (e.g. health conditions or diseases), types of research (e.g. basic or clinical) and/or other planned initiatives (e.g. workforce or research integrity).

The choice to focus on strategic planning was also guided by the fact that it is known from social science that strategic planning is a well-established practice within public organisations worldwide [17, 18]. This would enable us to ensure comparability of information on modalities of decision-making in research planning across organisations from different countries.

### Selection of public organisations

We created a list of public funders of health research, drawing from a previous study in which the authors identified 55 public and philanthropic organisations and listed them according to their annual expenditure on health research [16]. In order to strike a balance between learning about the practices of health research funders, and keeping data collection feasible and manageable, we restricted our sample to two organisations per country, with health research budgets of more than 200 million USD annually. In doing so, we identified a manageable subsample of 35 organisations having the greatest potential influence on research agendas, both locally and globally, and representing different health research systems in different countries.

We based our overview on publicly available information and restricted our sample to those organisations with published strategic research plans in English, French, Italian or Spanish (Additional file 1).

### Information search and abstraction

Since we expected processes to vary across organisations, we did not use guidelines or best practices for strategic planning, which allowed us to document a wide range of experiences. As mentioned earlier, we based this overview on the collection of publicly available information by means of a conceptual framework and an information abstraction form (Box 1, Additional file 1).

We based the conceptual framework on Walt and Gilson’s policy analysis model [19] and the information that could actually be retrieved after an initial assessment of the available information. The conceptual framework and the data abstraction form were conceived in an effort to (1) standardise the search for and collection of information across organisations, (2) render the collection process more transparent, and (3) make the retrieved information more understandable to readers.

Three authors (CM, CF and MCE) performed the review of information and the compilation of the form independently, with differences of opinion resolved by discussion. Information was collected in duplicate from 1 January 2019 to 31 July 2019. Before submitting the article, we updated the information by accessing and reviewing the official websites of the included organisations until 10 December 2019.

We searched for information that answered our questions by (1) browsing the funding organisations’ official websites and following links providing information about the organisations, e.g. Who we are, About us, Mission, Laws and statutes, Funding opportunities and other similar web pages, and by (2) identifying and reviewing strategic plans. When an organisation was composed of multiple sub-organisations, we limited our analysis to the strategic planning of the overarching organisation.

A second phase of research consisted of producing a profile for each organisation according to the data extraction form (Additional file 1). Bearing in mind that the results of this analysis could have been very general, we also used two organisations as case studies to provide more detailed examples of planning and implementing research priorities at the organisational level. We accessed and reviewed the official websites of the case study organisations until 14 April 2020. We did not contact organisations directly to obtain additional information. After collecting and analysing the information, we produced a narrative overview of our findings.

Box 1 Conceptual framework Organisation profile

 This section describes the funding organisation and its role and relationship with other overarching governmental bodies.

What are the contents of the strategic plan?

 This section examines the publicly available strategic plan of the funding organisation. The strategic plan is assumed to be the final outcome of the strategic planning process and includes the research priorities of the organisation. Depending on the mandate of the organisation, the research priorities are those related to research topics (for example, health conditions/diseases), types of research (for example, basic research, clinical research) and/or other planned initiatives within the mandate of the organisation (e.g. workforce, research integrity).

Regulatory basis

 This part seeks to understand if there is an official basis for strategic planning, for example, a law or a government document that establishes processes and actors for setting priorities.

What are the process and tools of strategic planning?

 This section seeks to describe the processes and tools for identifying the research priorities included in the strategic plan, including whether or not there are explicit mechanisms, criteria, instruments and information to guide and inform the process of strategic planning such as a research landscape analysis or a more structured experience of priority-setting.

Who are the actors involved?

 This section examines who the involved actors are in preparing the strategic plan; for example, who coordinates the process and who is involved in the process (e.g. clinicians, patients, citizens, researchers) and how the organisation relates with other entities in preparing the strategic plan.

## Results

### Included organisations

We included 11 public organisations with a publicly available strategic plan in English, Spanish, French or Italian (Additional file 1). There were two from the United States, two from France, and one each from the United Kingdom, Canada, Australia, Japan, Italy, Spain and Singapore. The mandates of the organisations were diverse – some had the task of funding research and other activities in support of health research, while others were involved in both funding and conducting health research (Table 1).

**Table 1:** Description of the selected organisations and of the development of their strategic plan

Organisation name	Country	Organisation description	Strategic plan	Regulatory basis for strategic planning	Main actors and development process
National Institutes of Health (NIH)	United States	National research agency; it is an operating division of the United States Department of Health and Human Services, organised in 27 theme-based institutes, centres and programme offices (ICOs); ICOs cover all fields of health research from basic research to applied research.	The NIH-Wide strategic plan Turning Discovery into Health Fiscal year 2016–2020	In 2015 and 2016, a legal basis for the overall NIH strategic planning was introduced; it requires NIH to produce a 5-year-wide strategic plan and provides some directions for selected contents (e.g. rare diseases) and the process to produce the NIH overall strategy (e.g. criteria to be considered, analysis of research landscape); it also stipulates that the NIH-coordinated strategy has to inform individual strategic plans of ICOs.	The first NIH-wide strategic plan was produced according to the new legal basis and published in 2016; the NIH Director and the Deputy Director developed a ‘framework’ for the strategic plan, designed with the purpose to identify major NIH cross-cutting areas of research and to set out principles (‘unifying principles’) to guide the NIH research effort. The development of the NIH-wide strategic plan involved extensive internal and external consultations throughout the process; internal consultation involved the ad hoc committee NIH-wide Strategic Plan Working Group with representatives of all 27 ICOs; the framework received input from the NIH governing: the Advisory Committee to the Director, composed of experts in the fields of research within the NIH mission, representatives of the research community (academia and private sector) and of the general public, and the National Advisory Councils of the IOCs; comments and suggestions on the framework were solicited in an external consultation process from a wide array of stakeholders such as representatives of patient advocacy organisations, professional associations, private hospitals or companies, academic institutions, government or private citizens (please see the 'case studies' paragraph below for more information on the actors and development process of NIH).
Medical Research Council (MRC)	United Kingdom	National funding agency dedicated to improving human health by supporting research in universities and hospitals, in MRC units, centres and institutes in the United Kingdom; the MRC is a committee body of the United Kingdom Research and Innovation (UKRI) alongside eight other committees, the ‘Councils’, representing various research sectors.	*Inserm 2020 Strategic Plan*	A recent law established the UKRI; the agency is funded by the Department for Business, Energy and Industrial Strategy with the responsibility of developing a national research strategy; the formation of UKRI is aimed at linking across disciplines and a better prioritisation of resources. The law stipulates that the MRC has to be part of the UKRI, as a committee body alongside the Councils, representing research sectors of science and humanities.	The MRC governing bodies develop the strategic plan; these are the Executive Chair, the Council, the Management Board and the Strategy Board In 2017, under the Higher Education and Research Act, the UKRI was established; according to the new law, in 2018, the MRC became a committee body of the UKRI alongside the Councils, representing research sectors of science and humanities. As described in the UKRI Framework document, UKRI became the responsible body for the development of a coherent UKRI strategy, balancing the allocation of funding across different disciplines; the Secretary of State has to approve the strategy. In addition, the MRC is required to develop a strategic plan that is coherent with the strategic objectives set by UKRI; the MRC strategic plan should be submitted for approval to the Board of UKRI, which is the governing body responsible for ensuring coherence among the Council plans and the UKRI strategy.
Institut national de la santé et de la recherche médicale (Inserm)	France	Inserm is a public scientific and technological institute that operates under the joint authority of the French Ministry of Health and the French Ministry of Research; it is organised in theme-based institutes and regional offices; the institute covers all fields of health research from basic research to applied research.	Inserm 2020 Strategic Plan	The law provides the basic requirements for the development and approval process of the plan; it names the governing bodies of Inserm, the stakeholders and the institutions to be involved.	The Administrative Council of Inserm (*Le Conseil d’administration*) should devise the research plan; it is composed of internal and external stakeholders and chaired by the President of the Institute; internal stakeholders are representatives of researchers, technicians and administrative staff of the Institute; a wide array of external stakeholders are involved in the process, including representatives for government (ministries of health, education, research, industry, budget), senior officials from research, healthcare organisations, academia and world of work, experts in economics, social sciences and biomedicine fields, and public health researchers. The Scientific Advisory Board (*Le Conseil scientifique*) provides input to the research plan; it includes internal stakeholders nominated by the staff of Inserm and members nominated by the Ministry of Health and Ministry of Research; in the plan, it is stated that the strategic priorities are aligned with France Europe 2020, the national strategic agenda for research transfer and innovation as well as with the national health plan and thematic national plans (national plan for rare diseases, cancer, neurodegenerative diseases); some priorities of the strategic plan are also from input of the Ministry of Health (e.g. genome medicine, programme to control bacterial resistance to antibiotics, translational and clinical research); the plan is signed off by the Ministry of Health and the Ministry of Research.
United States Department of Defense – Congressionally Directed Medical Research Programs (DoD-CDMRP)	United States	DoD-CDMRP is located within the United States Army Medical Research and Development Command; it funds a variety of theme-based health research programmes; the majority of DoD funds for health research are executed through the funds for CDMRP.	DoD CDMRP has strategic plans specific for each research programme	Currently, there is not an official legal basis for strategic planning; CDMRP recently reviewed the management of the research programmes and produced strategic plans upon the recommendations of the ad hoc Committee from the National Academies of Sciences, Engineering and Medicine, which were assembled at the request of the Senate. The Committee recommended the development of a strategic plan that should start from an analysis of a funding landscape across diverse agencies and organisations, should identify short-term and long-term research needs and opportunities, and find ways to coordinate with research priorities of other United States organisations.	Each CDMRP research programme has its own investment strategy; in order to devise the research strategy, the manager of each CDMRP programme organises a ‘vision-setting’ meeting, which defines investment strategy and award mechanisms. It involves the programmatic panel composed of scientists, clinicians, members of other federal agencies, experts in the specific health area and consumers (patients, caregivers). Programmatic panel decisions are supported by information on current state of science and knowledge gaps collected by the programme manager (literature review, consultation with other agencies and of the Federal RePORTER database of United States research projects).
Canadian Institutes of Health Research (CIHR)	Canada	CIHR is the health investment agency of the Government of Canada; it supports from fundamental to biomedical to applied research; it is composed of 13 theme-based institutes.	CIHR strategic plan 2014–15/2018–19	While there is no formal requirement for the publication of a strategic plan, CIHR produces and publishes a multi-year strategic plan on the official website. The law provides names of the governing bodies involved in the process for developing the strategic goals of the organisation. It requires the CIHR to submit, to the Ministry of Health, an annual report on activities in that fiscal year and its strategic directions and goals, with financial statements.	The CIHR governing bodies devise the strategic plan: the Governing Council, the Science Council and the Executive Management team. The Governing Council is composed of the president of CHIR, the Deputy Minister of the Department of Health (both non-voting members) and 18 members from academia and research institutes; it has a chair and vice-chair. The Science Council is chaired by the President and composed of scientific Directors of all 13 institutes and Directors/Chiefs of other CIHR governing bodies. The current strategic plan is based on the previous plan of 2009, when a national external consultation of research community was undertaken; in the plan, it is stated that a research landscape analysis was undertaken to support decision-making.
National Health and Medical Research Council (NHMRC)	Australia	It is the health research funding agency under the authority of the Australian Government Ministry for Health and Ageing.	National Health and Medical Research Council (2019); NHMRC Corporate Plan 2019–20	There is an official legal basis for the development of the strategic plan of the organisation. The legal framework defines some elements of the process, including naming the governing bodies of the organisation as well as those who are allowed to participate in the process.	Under the NHMRC Act, the Chief Executive Officer (CEO) sets major national health issues likely to arise during the 4-year period covered by the plan. The CEO devises the strategy in consultation with the Minister and the NHMRC governing bodies, namely the Council and the Research Committees; members of the Council are external stakeholders, including healthcare professionals, a person with expertise in consumer issues and an expert in Aboriginal and Torres Strait Islander health needs; the Research Committee consists of experts in various research fields and healthcare professionals, a consumer representative and a member of the Australian Health Ethics Committee. By law, the CEO has the power to establish the Working Committee to help carry out NHMRC functions; to this end, the Community and Consumer Advisory Group was established, a Working Committee with the functions to provide advice on health matters and on health and medical research matters from a consumer and community perspective; it is comprised of 11 consumers and community leaders in Australia; the Ministry of Health provides guidance on NHMRC’s strategic priorities and approves or revises the plan.
Centre National de la Recherche Scientifique (CNRS)	France	CNRS is a public multidisciplinary research institution under the authority of the French Ministry of Higher Education and Research; it conducts research in all areas of science, technology and society; research areas are grouped into 10 scientific institutes.	Éléments de prospective	There is an official legal basis for the strategic plan. The law defines the process for development of the strategic plan, including naming the governing bodies of the organisation as well as those who are allowed to participate in the process.	The Administrative Council (*Le Conseil d’ Administration*) is the CNRS governing body that devises the research plan; the Administrative Council is chaired by the President of the institute and is composed of internal and external stakeholders. Internal stakeholders are represented by research, technical and administrative staff of the Institute; external stakeholders are representative from the Government (ministries of health, education, research, and finance) from research organisations, academia and world work; there are also experts in the economics and social sciences fields and in the field of science and technology. The Scientific Advisory Board (*Le Conseil Scientifique*) provides input; it is composed of internal and external stakeholders; among the external stakeholders, eight members are non-French scientists.
Japan Society for Promotion of Science (JSPS)	Japan	It is an independent funding organisation established with the purpose of contributing to the advancement of science in the fields of the natural, biomedical, social sciences and humanities fields; it funds a wide spectrum of Japan’s scientific and academic research programmes.	JSPS 2017–2018	Not reported	The JSPS’s overall programme is set by internal leadership with the support of the Research Center for Science Systems; within the Center, eight programme groups are established, composed of scientists from various research areas selected every 3 years to ensure gender equity and balance among research institutions. The recommendations for research of the Research Center for Science Systems are based on the information collected through local and global research activities and analysed by the Center for Global Science Information and the Center for Science Information Analysis.
The Italian Ministry of Health (MoH)	Italy	The MoH is the main institution responsible for public health at the national level; within the MoH, the Directorate of the Health Research and Innovation has functions related to promotion and coordination of health research, including the planning and the management of funds for health research.	Programma Nazionale della Ricerca Sanitaria PNRS 2017–2019	There is an official legal basis for the strategic plan; the legal framework defines the process for developing the plan, including naming the committee and those who are allowed to participate in the process; it identifies the types of research interest (mainly clinical and health service research) and the award mechanisms; it specifies that the National health plan should be considered in developing the research plan.	The MoH takes the lead in health research planning through the 3-year national health research programme; the MoH develops the national health research programme in consultation with the Health Research Section of the Health Technical Committee whose members are nominated by Decree of the Minister of Health. The Health Research Section consists of internal stakeholders of the MoH; external stakeholders are nominated by the MoH, Ministry of Research and Ministry of Foreign Affair and by the Standing Conference on the Relations between the State, the Regions and the Autonomous Provinces; they are scientists and recognised experts in the field of health research; representatives of research agencies and institutions, which are entitled to receive MoH funding (e.g. the Italian National Institute of Health and the National Institutes for Scientific Research -research and healthcare provider institutions), might also be invited to participate at the meetings of the Health Research Section to provide their perspectives. The national health research programme is then adopted by the MoH in agreement with the Standing Conference on the Relations between the State, the Regions and the Autonomous Provinces.
Instituto de Salud Carlos III (ISCIII)	Spain	ISCIII is a public national organisation that funds and carries out health research; it is organised in centres, units, schools, and foundations and is under the authority of the Ministry of Economy, Industry, and Competitiveness through the Secretary of State for Research, Development and Innovation.	Planes Estatales de Investigación Científica, Técnica y de Innovación 2017–2020	In 2011, a legal framework for the investments in science and innovation was introduced; it aims to ensure accountability and responsiveness to science and societal needs of research organisations; it establishes the ‘State Plans’ as planning tools of the administration of the State for the implementation of the national government strategic actions. The law names the responsible organisations for devising both the national research strategy and the State Plan.	ISCIII manages the health research priorities as they are set out in the Strategic Action for Health (*Acción estratégica en Salud*) within the programmes of the State Plan 2017–2020 for Science, Innovation and Technology, in turn aligned to the National Spanish Strategy for Science, Innovation and Technology (*Estrategia española de ciencia y tecnología y de innovación* 2013–2020). By law, the Secretary of State for Research, Development, and Innovation of the Ministry of Finance (El Ministerio de Economía, Industria y Competitividad) takes the lead in developing the State Plan; the Advisory Committee for Science Technology and Innovation (*Consejo Asesor de Ciencia, Tecnología e Innovación*) within the Ministry of Science and Innovation provides advice. The development of the State Plan 2017–2020 involved experts from academia, industry, scientific societies and research organisations as well as experts in development of policies for research and development at national and international level and those responsible for programming investments of the General State Administration. The newly created Agency of Research (*Agencia, Estatal de Investigacion*) also participated; its main responsibility, however, is the financing and supervision of some of the programs of the State Plan, including the Strategic Action for Health.
Singapore National Medical Research Council (NMRC)	Singapore	It is one of the major government funders of biomedical and health research, under the authority of the Ministry of Health.	Research, Innovation and Enterprise 2020 plan	Not reported	The NMRC refers to the strategic plan developed by the National Research Foundation a department within the Prime Minister’s Office. In the field of health and biomedical sciences, the strategic plan focuses on five therapeutic areas of focus: cancer, cardiovascular disease, metabolic disease, infectious disease, and neurological and sense disorders based on factors such as disease impact, scientific excellence in Singapore and national needs NMRC establishes task forces for developing disease-based research strategy; these are made publicly available with a description of the priority-setting exercises; research priorities are used to inform NMRC fund grants (see Case Study).

### The strategic plan: format and content

The strategic plans varied in format (Additional file 1). While some organisations indicated broad lines of research, others structured their strategic plan in a complex hierarchy with high-level priorities connected to goals and sub-goals. In some cases, indicators, or menus of indicators, were added to monitor progress of the planned work and/or assess the impact of the research. In some research plans, the type of research funding (e.g. responsive, commissioned, research training) and budget were explicitly linked to research priorities.

With regard to content, some organisations focused their strategy on supporting the production of new knowledge of specific diseases or conditions. Others prepared a comprehensive strategy to support different functions of the health research system, such as producing knowledge, sustaining the workforce and infrastructure, developing policies for research integrity and conceiving processes for making more informed decisions. Some strategic plans briefly described the research environment at the national, organisational or programme level. One organisation described the process used to develop health research priorities.

### Strategic planning

#### Regulatory basis

Most of the organisations are legally required to present a multi-year strategic plan or at least annual research priorities. In addition, legislation sets rules and procedures by covering subjects such as the actors to be involved, the documents to be consulted and the format of the strategic plan document to be adopted. In some cases, legal provisions indicate areas and/or types of research to be funded (Table 1).

#### Actors

Commonly, the main actors are the top-level policy-makers of the organisations. A spectrum of external stakeholders from multiple sectors may be involved and their participation varies across organisations. External stakeholders can be members of academia or government research agencies, or industry professionals and policy-makers. Most frequently, they have a membership role in organisational governing bodies (boards and committees) (Table 1).

The government maintains a role in shaping the strategic plan to various extents in different organisations. This may involve producing nationwide strategic plans for research that the organisations have to adopt or align to, directing attention to specific research priorities or types of research, having representatives in the governing bodies of the organisations and retaining the power of final approval of the organisations’ strategic plans (Table 1). Other actors involved are overarching government agencies, which play a role in managing or coordinating the research plan at the national level. Examples of this are the Spanish National Research Agency and United Kingdom Research and Innovation (UKRI). When this study was being conducted, the latter had just been established and been given the role of developing a coherent national research strategy.

The participation of civil society in governing bodies, temporary committees or consultation exercises was far less common. There are representatives of the public in the advisory bodies of the National Institutes of Health (NIH; e.g. the Advisory Committee to the Director).

The Chief Executive Officer of the National Health and Medical Research Council (NHMRC), acting under the terms of the NHMRC Act, established the Community and Consumer Advisory Group. This is a working committee whose function is to provide advice on health questions and health and medical research matters, from consumer and community perspectives. Most notably, the United States Department of Defense – Congressionally Directed Medical Research Programs (DoD-CDMRP) involve consumers (patients, their representatives and caregivers) at all levels of the funding process, from strategic planning to the peer-review process of research proposals. Organisations also have external consultation exercises, in which the target audiences and mechanisms implemented vary (Table 1).

#### Process

In order to illustrate the interactions between different actors, we identified two broad categories of organisation. The first comprises those organisations that develop their own plans with a certain degree of independence. Government and legal provisions might provide some direction. In this group are the NIH, the Institut national de la santé et de la recherche médicale (Inserm), the Italian Ministry of Health (MoH), the NHMRC, the Canadian Institutes of Health Research (CIHR), the Medical Research Council (MRC), the DoD-CDMRP, the Centre National de la Recherche Scientifique (CNRS) and the Japan Society for the Promotion of Science (JSPS) (Table 1).

The second category is made up of those organisations whose research planning derives from the strategic plan of an overarching entity. In this group are the Instituto de Salud Carlos III (ISCIII), the National Medical Research Council (NMRC) and the MRC. Both categories are represented in the case studies below.

An example of the first category from the United States is the 5-year strategic plan, NIH-Wide Strategic Plan, Fiscal Years 2016–2020: Turning Discovery Into Health, developed by the NIH at the request of Congress. Legislation provides direction on some criteria for setting priorities in the plan, but it is the NIH Director who develops it in consultation with internal (Centres, Institutes and Offices) and external stakeholders (see the NIH case study).

In Australia, the Chief Executive Officer of the NHMRC identifies major national health issues likely to arise during the 4-year period covered by the plan and devises the strategy in consultation with the Minister for Health and the NHMRC governing bodies. The Minister provides guidance on the NHMRC’s strategic priorities and approves or revises the plan. In Canada, the governing bodies of the CIHR are responsible for devising the strategic plan. The Deputy Minister of the Department of Health participates as a non-voting member of one of the governing bodies.

The common characteristic of the second category is that the process of strategic planning derives from one or more overarching entities. This means that the strategic plans of the organisations are informed to various extents by the research programmes of such an entity or entities. In some cases, there is a main institution with research coordination and/or management roles at the national level. For example, in Spain, in order to inform funding grants, the ISCIII adopted the research priorities set out in the Strategic Action for Health included in the State Plan for Science, Innovation and Technology 2017–2020*.* This plan, elaborated by the Government Delegated Committee for the Policies for Research, Technology and Innovation (*la Comisión Delegada del Gobierno para Política Scientífica, Tecnológica y de Innovación*), in cooperation with the Ministry of Fianance, is aligned with the four strategic objectives of the Spanish Strategy for Science, Technology and Innovation 2013–2020. The newly established Spanish State Research Agency (*Agencia Estatal de Investigacion*) also participated in the development of the State Plan. However, its role is mainly in monitoring the plan’s funding, including ISCIII funding for the Strategic Action for Health.

UKRI, sponsored by the Department for Business, Energy and Industrial Strategy, is the body responsible for the development of a coherent national research strategy that balances the allocation of funding across different disciplines. In 2018, the MRC became a committee body of UKRI, alongside eight other committees, called ‘Councils’, which represent various research sectors. The MRC is required to develop a strategic plan that is coherent with the strategic objectives set by UKRI. This plan must be approved by the UKRI Board, the governing body responsible for ensuring that Council plans are consistent with the UKRI strategy.

In Singapore, the NMRC refers to the strategic plan developed by the National Research Foundation, a department within the Prime Minister’s Office. The NMRC has a well-described system for incorporating national priorities into the organisation’s research plan (see the NMRC case study).

With regard to the information, sources of information, criteria and mechanisms used to shape decisions, the included organisations were less explicit. Most commonly, organisations introduced health research priorities with an overview of major general advancements in biomedical research or a catalogue of organisational activities and a research portfolio.

A small number of organisations presented a brief situational analysis of the health and health research sectors. In these cases, the scope and nature of the presented information varied from one organisation to another (Additional file 1).

For example, the NIH-Wide Strategic Plan contains a brief summary of the state of research at the organisational level. The plans of each DoD-CDMRP health research programme present a summary of both the current health and health research landscapes at the national level.

Other organisations stated that the plan had been supported by information analysis of the research field, but they did not report explicitly on this work.

### Case studies

#### The National Institutes of Health (NIH)

The NIH is an operating division of the United States Department of Health and Human Services whose mission is to improve public health by conducting and funding basic and translational biomedical research. It is made up of 27 theme-based Institutes, Centers and Offices, each of which develops an individual strategic plan [20].

The first 5-year strategic plan, NIH-Wide Strategic Plan, Fiscal Years 2016–2020: Turning Discovery into Health, was prepared at the request of Congress and published in 2016 [21]. The legal framework stipulates that the NIH-coordinated strategy will inform the individual strategic plans of the Institutes and Centers. In addition, it provides some direction regarding content and the process to be adopted for generating the overall NIH strategy [22, 23]. For example, it sets out specific requirements for the identification of research priorities. These include “*an assessment of the state of biomedical and behavioural research*” and the consideration of “*(i) disease burden in the United States and the potential for return on investment to the United States; (ii) rare diseases and conditions; (iii) biological, social, and other determinants of health that contributes to health disparities; and (iv) other factors the Director of National Institutes of Health determines appropriate*” [23]. The NIH Director is also required to consult “*with the directors of the national research institutes and national centers, researchers, patient advocacy groups and industry leaders*” [23]. To fulfil the request of Congress, the NIH Director and the Principal Deputy Director initiated the process by creating a draft ‘framework’ for the strategic plan. This framework was designed with the purposes of identifying major areas of research that cut across NIH priorities and of setting out principles to guide the NIH research effort (‘unifying principles’).

The development of the NIH-Wide Strategic Plan involved extensive internal and external consultations throughout the process. Consultees included the ad hoc NIH-Wide Strategic Plan Working Group, composed of representatives of all 27 Institutes, Centers and Offices, the Advisory Committee to the Director, which is an NIH standing committee of experts in research fields relevant to the NIH mission, and representatives of the research community (from academia and the private sector) and the general public. The framework was also presented at meetings with the National Advisory Councils of the Institutes and Centers.

In addition, the framework was disseminated to external stakeholders for comments and suggestions, which were solicited via a series of public webinars and through the initiative Request for Information: Inviting Comments and Suggestions on a Framework for the NIH-Wide Strategic Plan. In this case, a web-based form collected comments and suggestions on a predefined list of topic areas from a wide array of stakeholders representative of patient advocacy organisations, professional associations, private hospitals and companies, academic institutions, government and private citizens [24–27]. A report on the analysis of the public comments is publicly available [27].

#### The National Medical Research Council (NMRC)

The NMRC is the organisation that has the role of promoting, coordinating and funding biomedical research in Singapore [28]. It has developed its own research strategy by adopting the research priorities indicated by the national research strategy in the domain of health and biomedical sciences [29].

The national research strategy is the responsibility of the National Research Foundation, a department of the Prime Minister’s Office. It defines broad research priorities relating to various areas of research identified as ‘domains’. Within the health and biomedical sciences domain, five areas of research have been proposed with input from the Ministry of Health and the Health and Biomedical Sciences International Advisory Council. These are cancer, cardiovascular diseases, infectious diseases, neurological and sense disorders, diabetes mellitus and other metabolic/endocrine conditions. Criteria for selection of the areas of focus were “*disease impact, scientific excellence in Singapore and national needs*” [29].

The approach of NMRC to implementing the national research strategy at organisational level involves the establishment of ‘task forces’, i.e. groups of experts, with the role of defining the specific research strategy for each of the five areas of focus. Each task force provides documentation of research recommendations and methods used to prioritise research topics [30].

For example, the Neurological and Sense Disorders Task Force identified sub-areas of research^1^ after analysing the local burden of neurological and sense disorders as well as considering factors such as local scientific expertise and research talent, ongoing efforts in neurological and sense disorders, industry interest, and opportunities for Singapore. As part of the effort, input was also solicited from the research community and policy-makers. This research prioritisation exercise served for both the NMRC grant scheme and a 10-year research roadmap [31].

## Discussion

Our study is the first to report on the processes used by a set of large national public funders to develop health research strategic plans. In line with findings from public management literature [16, 17], we found that the formulation of a strategic plan is a well-established practice in shaping research agendas across international settings and it is a legal requirement for the majority of the organisations we studied.

We were able to reconstruct the process for developing the strategic plan by identifying the main actors involved and how they are connected. A complex picture emerges, in which multiple interactive entities and forces, often organised in a non-linear dynamic, appear to shape the research plans. In general, an organisation has to take into account legislative provisions, government directives, national overall research plans, national health plans and specific disease area plans. In some cases, it has to consider ‘institutionalised’ allocation of resources across organisations’ sub-entities (institutes, centres and units), which are historically associated with a particular disease or type of research.

On the other hand, we found little documentation of the decision-making mechanisms and information used to inform decision-making. There were, for example, few references to health research needs, research capabilities, the sources of information consulted, and the principles and criteria applied. This despite the increasing attention being paid nationally and internationally to the need for an explicit evidence-based or rational approach to setting health research priorities, particularly in the light of current economic constraints [3, 32, 33]. Given the complexity of the influences of different parties and factors, the governance of the health research sector would benefit from a traceable knowledge-based process of strategic planning, similar to that advocated for the health sector [34].

We found, however, evidence of an increasing interest in improving ways to establish research priorities at the organisational level. For example, NIH has brought forward the Senate request to develop a coordinated research strategy by including, in the strategic plan, the intention to further improve the processes for setting NIH research priorities and to optimise approaches to making informed funding decisions [21].

Recently, the DoD-CDMRP, the second largest funder of health research in the United States, reviewed its research management practices upon the recommendations of an ad hoc committee of the National Academies of Sciences, Engineering and Medicine. In the area of strategic planning, the committee recommended an analysis of the funding landscape across different agencies and organisations, the identification of short- and long-term research needs, and harmonisation with the research priorities of other organisations [35].

In its strategic plan, the JSPS has placed particular emphasis on the development of research-on-research capacity and infrastructures to analyse the research landscape at organisational, national and international levels in order to ensure that funding decisions are evidence based [36].

The allocation of sufficient resources to develop the infrastructure and technical expertise required for collection, analysis and dissemination of a portfolio of relevant data should be considered a necessary step when a funding organisation or country decides to implement standardised approaches for strategic planning and priority-setting.

Additionally, from the perspective of health research as a system, data collection and analyses should not be limited to ‘what is funded’, but should also include ‘who is funded and where’, and be linked to research policies and their long-term outcomes. The benefit of such an approach is not limited to the prevention of unnecessary duplication of research. Support would also be provided for producing formal mechanisms to coordinate research effort across research entities, within and among countries. Collaborations with other non-profit as well as for-profit organisations would be promoted and the capacity for research would be created and strengthened where necessary.

A number of resources and initiatives in this field already exist at organisational and national level. For example, the NIH has the Research Portfolio Online Reporting Tools, a public repository of data and other tools from NIH research activities [37]. This repository is linked to Federal RePORTER, an infrastructure that makes data on federal investments in science available. In the United Kingdom, the Health Research Classification System performs regular analysis of the funding landscape of United Kingdom health research to support monitoring, strategy development and coordination [38].

At the international level, there is ongoing global work to shape evidence-based health research decisions and coordination. In 2013, the WHO Global Observatory on Health R&D was established “*in order to monitor and analyze relevant information on health research and development,* […] *with a view to contributing to the identification of gaps and opportunities for health research and development and defining priorities* […] *and to facilitate the development of a global shared research agenda*” [33]. This effort has been coupled with a global call to action, which asks governments to create or strengthen national health research observatories and contribute to the WHO Observatory. Furthermore, the Clinical Research Initiative for Global Health, a consortium of research organisations across the world, has ongoing projects that will map clinical research networks and funding capacity and conduct clinical research at a global level [39].

A further key area that deserves comment is the engagement of stakeholders. In general, a spectrum of external stakeholders from multiple sectors is involved and the extent of this involvement varies across organisations. Decision-making processes commonly include people from government bodies, academia, research agencies and industry. However, we found that the participation of civil society, here represented by the intended beneficiaries of research such as health professionals, patients and their carers, remains limited. The fact that decision-making is still the domain of government officials and experts is an unexpected finding. There is a widespread consensus that the participation of a mix of stakeholders can improve the process of strategic planning. The logic behind this is that representatives of those who are affected by decisions can bring new information and perspectives and improve the effectiveness of the process [17, 32, 40]. Broader inclusion is desirable, both for granting legitimacy to strategic planning and for advancing equity in healthcare. Decisions on research priorities shape knowledge and, ultimately, they determine whether patients and their carers will have access to healthcare options that meet their needs [41].

Additionally, our study shows that the involvement of civil society is not only desirable but is also feasible. Organisations that support the participation of civil society have this practice firmly embedded in their governance, although it may be implemented in different ways.

### Strengths and limitations

A particular strength of our study is the innovative way in which we approached the disorienting complexity of whole-organisation planning cycle management. This allowed us to contribute to an understanding of the processes used by large public funders not only in English-speaking countries but also in France, Italy and Spain.

However, one potential limitation concerns the accuracy and completeness of the information. This drawback was imposed by both the unstructured nature of the information and its fragmentation across multiple webpages and legal and/or administrative documents. Nevertheless, we strove to ensure accuracy, consistency and a clear presentation of the relevant information by means of a conceptual framework and a data abstraction form. In addition, to guarantee the reliability of the data, two reviewers abstracted the information independently, before discussing it and reaching a consensus. The use of more accessible information, e.g. through single documents, would therefore be advisable to improve accountability and transparency. This would also be of particular importance for exchanging knowledge and promoting research in the specific field of research governance.

In addition to the limitations imposed by the available data, there is a potential limitation in the methodology of the study. In conducting our research, we decided to rely only on publicly available information and we did not ask organisations for further details. Consequently, we may have missed some actions and drawn an incomplete picture of the organisations presented. Our strategy was based on the assumption that, if a strategic plan existed, both it and a description of its associated decision-making process would be present in the public domain, given that transparency in decision-making is an acknowledged element of good public organisation governance [42]. We would therefore counter that the process should be more transparent and should address, in particular, the criteria and information used to support decision-making.

In addition, it was not possible to ascertain in detail how processes actually took place. For example, engaging external stakeholders, such as representatives of civil society, is a key feature of the organisations included in the study but we do not know whether this engagement was meaningful or simply granted legitimacy to leadership decisions.

Furthermore, by limiting our inclusion criteria to organisations with strategic plans publicly available in English, French, Spanish and Italian, we excluded two German organisations (the German Research Foundation and the Bundesministerium für Bildung und Forschung – the Federal Ministry of Education and Research) and two Chinese bodies (the National Natural Science Foundation of China and the Ministry of Health). These organisations could have been included on the basis of their health research budgets. While it is unlikely that these bodies from two countries with similar health research systems have practices that would have changed our conclusions, it would nevertheless be useful in the future to acquire information regarding their experiences in this area.

### Future research

Having considered the abovementioned limitations, we recommend that qualitative research be conducted to further validate our findings by complementing the information presented here with data gathered from key informants within each organisation. We also suggest that the study be extended to include other organisations and countries. Additional research should also expand on our study by more deeply exploring the perspectives of the members of external stakeholder bodies regarding their involvement in strategic planning within each organisation. Making this information accessible would benefit those funder organisations who wish to both increase public engagement in health research decision-making and make it more meaningful.

It would also be interesting to explore whether and why funder organisations are influenced by the research plans of other organisations (including academic, advocacy and international bodies) within and among countries, and whether they have formal mechanisms in place to coordinate with other such organisations. This information would be of use in guiding research coordination policies, with the aim of avoiding duplication of effort and identifying not only gaps in research but also overlapping interests and opportunities for partnerships.

## Conclusion

Our study illustrates the variety of the processes adopted in developing strategic plans for health research in the international setting. A complex picture emerges in which multiple interactive entities appear to shape research plans. Although we found documentation of the actors involved in the processes, much less was available on the mechanisms, information, criteria and tools used to inform decision-making.

Given the complexity of the influences of different parties and factors, both funding organisations and health sector governance would benefit from a traceable knowledge-based process of strategic planning. The benefits of such an approach are not limited to demonstrating responsible budget stewardship as it would also provide opportunities to respond to research gaps and healthcare needs and to move more effectively from basic to translational research.

## Supplementary information

**Additional file 1.** Data abstraction form. (a) Table of the included and excluded organisations with reasons; (b) table of the included organisations with budget; (c) the organisations’ profiles according to the fields of the conceptual framework; and (d) full list of the consulted references and web pages for each organisation’s profile.

## Data Availability

All data generated or analysed during this study are included in this published article in Additional file 1.

## Abbreviations

HICs: High-income countries
UKRI: United Kingdom Research and Innovation
NIH: National Institutes of Health
NHMRC: National Health and Medical Research Council
DoD-CDMRP: U.S. Department of Defense - Congressionally Directed Medical Research Programs
Inserm: Institut national de la santé et de la recherche médicale
MoH: The Italian Ministry of Health
CIHR: Canadian Institutes of Health Research
MRC: Medical Research Council
CNRS: Centre National de la Recherche Scientifique
JSPS: Japan Society for Promotion of Science
ISCIII: Instituto de Salud Carlos III
NMRC: Singapore National Medical Research Council

## Footnotes

1 Neurodegenerative diseases (vascular dementia and Parkinson’s diseases), neurodegenerative eye diseases (age-related macular degeneration and glaucoma), mental health disorders (depression) and neurotechnology.
